# Clinical Presentation and Conservative Management of Tympanic Membrane Perforation during Intrapartum Valsalva Maneuver

**DOI:** 10.1155/2010/856045

**Published:** 2010-03-02

**Authors:** Jonathan D. Baum, Meghan I. Rattigan, Eric Scott Sills, Anthony P. H. Walsh

**Affiliations:** ^1^Department of Obstetrics and Gynecology, Jersey Shore University Medical Center, 1944 State Route 33, Second Floor, Neptune, NJ 07753, USA; ^2^Department of Obstetrics and Gynaecology, School of Medicine, Royal College of Surgeons in Ireland, Dublin, Ireland

## Abstract

*Background.* Tympanic membrane perforation may occur when ear pressures are excessive, including valsalva maneuver associated with active labor and vaginal delivery. A pressure differential across the eardrum of about 5 psi can cause rupture; the increased intraabdominal pressure spikes repeatedly manifested by “pushing” during second-stage labor easily approach (and may exceed) this level. *Material and Method.* We describe a healthy 21-year old nulliparous patient admitted in active labor at 39-weeks' gestational age. *Results.* Blood appeared asymptomatically in the left ear canal at delivery during active, closed-glottis pushing. Otoscopic examination confirmed perforation of the left tympanic membrane. Complete resolution of the eardrum rupture was noted at postpartum check-up six weeks later. *Conclusion.* While the precise incidence of intrapartum tympanic membrane rupture is not known, it may be unrecognized without gross blood in the ear canal or subjective hearing loss following delivery. Only one prior published report on tympanic membrane perforation during delivery currently appears in the medical literature; this is the first English language description of the event. Since a vigorous and repetitive valsalva effort is common in normal vaginal delivery, clinicians should be aware of the potential for otic complications associated with the increased intraabdominal pressure characteristic of this technique.

## 1. Background

Rupture of the tympanic membrane can result from any pressure or stress exerted on the ear. The vigorous and repetitive valsalva efforts of active labor can yield internal ear pressures that exceed the safe threshold for tympanic membrane integrity, causing intrapartum injury to the eardrum. Intrapartum tympanic membrane rupture appears to be encountered with low frequency, although under-reporting could be a result of inadequate clinical familiarity and minimal awareness of the condition.

## 2. Clinical Presentation

A 21-year-old nonsmoking nullipara presented at 39-weeks' gestation in active labor. Her prenatal course was unremarkable; results from all routine prenatal laboratory tests were normal. The patient had no prior ear infections, tympanoplasty or other surgery, and her baseline evaluation identified no deficit in hearing acuity. With the exception of prenatal vitamins, she took no regular medications. Social history was negative for diving, mountainclimbing, skydiving or other activities at barometric pressure extremes. Intravenous oxytocin was not indicated for labor augmentation. Fetal status remained reassuring and the patient achieved full cervical dilation and effacement within 90 min of admission. Neither neuraxial anaesthesia nor intravenous narcotics were required for pain management. Upon confirmation of full cervical dilation, the patient began closed-glottis pushing simultaneous with uterine contractions. Following a 32-minute second stage of labor, she delivered a viable male infant over a mediolateral episiotomy. Five and ten minute Apgar scores were 9 and 9; birth weight was 3291 g. The placenta was delivered spontaneously and intact. The episiotomy was repaired routinely. Intrapartum blood loss was approximately 300 mL.

Mother and baby did well following delivery. While the mother had no complaints in the postpartum period, she was noted to have several new small petechiae on her face and blood in her left ear about one hour after delivery. The right ear appeared grossly normal and the patient denied placing anything in the ear. However, otoscopic examination of the left external auditory canal revealed minimal dark blood and 1 cm superficial thrombus blocked a clear view of the eardrum. No active bleeding was observed and the patient remained afebrile. The patient reported no ear pain and denied any reduction in hearing sensitivity from either ear. Gentle extraction resulted in removal of the clot, revealing a 2-3 mm perforation in the inferior aspect of the left tympanic membrane. Otic lavage with bulb-syringe was not performed. Consultation with an ophthalmologist confirmed left tympanic membrane perforation. The contralateral tympanic membrane was intact. Cotton packing was placed in the ear canal and the left tympanic membrane was allowed to heal; antibiotics were not prescribed. At the postpartum follow-up exam six weeks later, the patient had no complaint and no additional ear bleeding was reported. She had no ear pain or hearing deficit, and the left eardrum appeared grossly normal. The patient was counseled to treat future ear infections promptly, to seek treatment immediately should any ear discharge be noted, and to avoid insertion of any object into the ear to clean it.

## 3. Discussion

The second stage of labor begins with full dilation. Expulsion of the fetus is accompanied by several physiologic events associated with remarkable changes in intra-abdominal pressure. The “forces by which the child is expelled” classically include voluntary (straining or bearing down) and involuntary (uterine contraction) components [1]. During vaginal delivery, the laboring woman is often encouraged to inhale deeply, close her glottis, and contract her abdominal muscles to generate greatly increased intra-abdominal pressure [2]. The transient but repeated pressure elevations can also affect other organs including the eye [3] and lung [4]. A substantial rise in middle ear pressure also occurs, and may result in tissue damage if not equilibrated gradually [5].

In humans, the tympanic membrane can withstand only limited pressure differentials and may rupture when this pressure exceeds 35 kPa (5 psi) [6]. While this pressure threshold is different for individual patients, at 100 kPa (14 psi) almost all eardrums will be ruptured [7]. The most common cause of ear barotrauma is high-altitude air travel [8] but modern pressurized cabins have virtually eliminated this risk. The effect is also operant during rapid ascent while scuba diving [9] and if transcanal pressure is not equalized, perforation of the tympanic membrane may occur [10]. In our case, the patient appears to have generated a pressure gradient during closed-glottis pushing similar to that registered during quick ascent in an unpressurized aircraft cabin or while scuba diving, but intra-abdominal pressure was not directly measured during delivery for our patient. Previous work has reported the average intra-abdominal pressure associated with “bearing down efforts” of second-stage labor to be about 25 kPa [11]. 

Given the largely unavoidable urge to valsalva vigorously and the severe spike in middle ear pressure associated with labor, it is somewhat surprising that tympanic membrane rupture is not encountered more often during delivery. However, pregnant patients can be counseled about spontaneous pushing techniques (open glottis pushing while breathing out) in the second stage of labor. This approach has been associated with subjectively “more effective pushing”, shorter second stage labor and improved neonatal outcome [12]. While an eardrum perforated during labor may be uncomfortable, it typically heals on its own. Any hearing loss that accompanies tympanic membrane perforation is usually transient, although patients should seek reevaluation if ear symptoms persist or worsen.

## 4. Conclusion

While the incidence of intrapartum eardrum perforation is unknown, our report is only the second published description of this event [13] and is the first such case to appear in the English medical literature. As this case illustrates, comanagement with an otolaryngologist is helpful for any pregnant or post-partum patient with blood observed in the ear canal, or whenever subjective change in hearing is noted. Notwithstanding the standardized role of the valsalva maneuver during second-stage labor [12], alternate pushing strategies should be considered whenever possible. While instrumental vaginal delivery (i.e., vacuum, forceps) reduces maternal effort during the second stage of labor, the risk of such intervention must be evaluated against the incidence of intrapartum tympanic membrane rupture, which is likely to be quite low.
